# Development of a Risk Stratification Model for Coronary In-Stent Restenosis Based on Clinical, Laboratory, and Procedural Factors: A Case–Control Study

**DOI:** 10.3390/jcm15145697

**Published:** 2026-07-21

**Authors:** Natalya Zemlyanskaya, Viktor Zemlyanskiy, Marat Aripov, Gulsum Mauletbayeva, Khaiyom Mahmudzoda, Said Abdullozoda, Gulmira Derbissalina

**Affiliations:** 1 Department of General Medical Practice with a Course of Evidence-Based Medicine, NJSC “Astana Medical University”, Astana 010000, Kazakhstan; mauletbaeva.g@amu.kz; 2 Interventional Radiology Department, CF “University Medical Center”, Astana 010000, Kazakhstan; 3 Interventional Cardiology Department, CF “University Medical Center”, Astana 010000, Kazakhstan; dr.aripov@gmail.com; 4 Department of Propaedeutics of Internal Diseases, SEI “Avicenna Tajik State Medical University”, Dushanbe 734000, Tajikistan; mahmudovkh@yandex.ru; 5 Department of Epidemiology named after Prof. Rafiev H.K., SEI “Avicenna Tajik State Medical University”, Dushanbe 734000, Tajikistan; saidxoja@gmail.com

**Keywords:** in-stent restenosis, coronary artery disease, percutaneous coronary intervention, risk factors, revascularization

## Abstract

**Background**: Coronary in-stent restenosis (ISR) remains a major limitation of percutaneous coronary intervention (PCI) with drug-eluting stents (DES), adversely affecting long-term outcomes. Most available prediction models rely on invasive procedural variables and have been developed predominantly in high-income populations, limiting their generalizability. This study aimed to identify independent predictors of coronary ISR and to develop and internally validate a clinically applicable risk stratification model based on routinely available clinical, laboratory, and procedural factors in a cohort of patients from Kazakhstan. **Methods**: In this retrospective case–control study, 910 patients with coronary artery disease (CAD) who underwent follow-up coronary angiography after PCI between January 2018 and July 2025 were included. The study comprised 455 patients with angiographically confirmed coronary in-stent restenosis and 455 patients without restenosis selected using a consecutive sampling approach. Clinical characteristics, laboratory parameters, echocardiographic findings, and angiographic data were analyzed. Independent predictors were identified using multivariable binary logistic regression. Model discrimination was assessed using receiver operating characteristic (ROC) curve analysis, and internal validation was performed using bootstrap resampling. **Results**: The mean age was 62.9 ± 8.9 years, and 75.2% of patients were male. Restenosis was independently associated with prior myocardial infarction (MI) (OR 2.20; 95% CI 1.65–2.80), type 2 diabetes mellitus (T2DM) (OR 2.60; 95% CI 1.93–3.47), and smoking (OR 1.40; 95% CI 1.01–1.89). Patients with restenosis demonstrated a less favorable inflammatory and metabolic profile, including higher NLR, MHR, atherogenic index, and TyG index (all *p* < 0.05). LVEF was significantly lower, while multivessel disease and the number of implanted stents was higher (*p* < 0.001). A risk stratification model incorporating T2DM, the number of implanted stents, MPV, neutrophil count, HDL-C, LVEF demonstrated good discrimination (AUC 0.828) and 74.4% accuracy. **Conclusions**: The proposed model demonstrated good discrimination and satisfactory internal validity with limited optimism after internal bootstrap validation. It may serve as a useful tool for patient risk stratification after PCI. External validation in independent cohorts is required before widespread clinical implementation.

## 1. Introduction

Coronary artery disease remains one of the leading causes of mortality and disability worldwide. By 2050, the global incidence, prevalence, and mortality of CAD are projected to reach 67.3 million, 510 million, and 16 million cases, respectively [1]. PCI with coronary stent implantation has become the standard method of myocardial revascularization for patients with obstructive CAD, improving symptoms, quality of life, and clinical outcomes [2]. Millions of PCI procedures are performed annually worldwide, and their number continues to increase [3]. In the Republic of Kazakhstan alone, approximately 32,000 PCI procedures are projected to be performed in 2025, emphasizing the growing importance of optimizing long-term outcomes after coronary intervention.

Despite substantial advances in drug-eluting stent technology, ISR remains an important cause of recurrent myocardial ischemia, repeat coronary revascularization, and adverse cardiovascular outcomes, occurring in approximately 10–20% of patients depending on lesion complexity and individual clinical characteristics [4,5,6]. The development of ISR is multifactorial, involving complex interactions among patient-related characteristics, procedural factors, and vascular biological responses [7]. In addition, demographic and ethnic differences in the prevalence of major cardiovascular risk factors may contribute to variability in ISR risk across different populations [8].

Although numerous prediction models for ISR have been proposed, no universally accepted model has been established. Most existing models rely on angiographic, procedural, or other invasive variables that are not routinely available during outpatient follow-up, limiting their applicability in routine clinical practice. Furthermore, the majority of these models have been developed in high-income countries, restricting their generalizability to other healthcare systems and patient populations [9]. Therefore, this study aimed to identify independent predictors of coronary in-stent restenosis and to develop and internally validate a clinically applicable risk stratification model based on routinely available clinical, laboratory, and procedural factors in a cohort of patients from Kazakhstan.

This manuscript was prepared in accordance with the STROBE (Strengthening the Reporting of Observational Studies in Epidemiology) reporting guidelines and the TRIPOD (Transparent Reporting of a multivariable prediction model for Individual Prognosis Or Diagnosis) Statement.

## 2. Materials and Methods

The study was conducted in accordance with the Declaration of Helsinki and approved by the Institutional Ethics Board of NJSC “Astana Medical University”.

### 2.1. Participants and Study Design

This retrospective case–control study included 910 patients with coronary artery disease who had previously undergone PCI with coronary stent implantation and were treated at the Heart Center, Corporate Fund “University Medical Center”, Astana, Kazakhstan, between January 2018 and July 2025. The center provides tertiary cardiovascular care to patients from all regions of Kazakhstan, making the study cohort representative of the national population undergoing PCI.

At the initial stage, participants were selected from 11,600 patients with coronary artery disease who were hospitalized during the study period and underwent coronary angiography, based on predefined inclusion criteria. Using a consecutive sampling approach, all eligible patients with angiographically confirmed in-stent restenosis were identified, resulting in a case group of 455 patients. The control group consisted of 455 patients without angiographically confirmed in-stent restenosis who met the same inclusion and exclusion criteria as the case group. Controls were selected from the same retrospective cohort and were frequency-matched to the case group to achieve comparable distributions of baseline demographic and clinical characteristics between the study groups. Individual patient matching was not performed.

Repeat coronary angiography was performed based on clinical indications (recurrent angina pectoris, positive ischemia testing, or acute coronary events) or at the discretion of the treating cardiologist, particularly in patients with technically complex index percutaneous coronary interventions (e.g., chronic total occlusions, bifurcations, multiple or long stents). Routine surveillance angiography in asymptomatic patients without clinical indications was not performed.

After completion of patient enrollment in July 2025, the study database was closed, and statistical analyses were subsequently performed.

### 2.2. Inclusion Criteria

Patients with a diagnosis of coronary artery disease, including stable angina pectoris, unstable angina, myocardial infarction, or ischemic cardiomyopathy, who had previously undergone PCI with coronary stent implantation.

### 2.3. Exclusion Criteria

Patients who underwent repeat coronary angiography less than 9 months after coronary stent implantation, those with previously implanted bare-metal stents (BMS), patients who had undergone coronary artery bypass surgery, individuals with diagnosed malignant tumors, and patients with chronic kidney disease stages 4 or 5.

### 2.4. Data Collection

During hospitalization, all patients underwent the standard diagnostic evaluation routinely performed at the Heart Center in accordance with the clinical guidelines of the Ministry of Healthcare of the Republic of Kazakhstan, including symptom assessment, medical history taking, physical examination, and laboratory and instrumental investigations. Variables for which data were available for all studied patients were selected for comparison. Only variables with complete data available for all included patients were analyzed; therefore, no imputation of missing data was performed. The final dataset comprised 72 variables, including 17 categorical and 55 continuous variables.

The TyG index was calculated using the following formula: ln [Triglycerides (mg/dL) × Fasting blood glucose (mg/dL)/2]. Triglyceride concentrations, originally expressed in mmol/L, were converted to mg/dL before calculation using a conversion factor of 88.57, whereas fasting blood glucose values were already expressed in mg/dL. The atherogenic index was calculated as (Total cholesterol − HDL-C)/HDL-C.

Angiographically, restenosis was defined as a narrowing of the coronary artery lumen at the stent site of ≥50%.

### 2.5. Statistical Analysis

Continuous variables were initially assessed for normality using the Kolmogorov–Smirnov test. Depending on the data distribution, differences between groups were evaluated using the independent-samples Student’s *t*-test or the Mann–Whitney U test. Normally distributed variables are presented as the mean ± standard deviation (SD), whereas non-normally distributed variables are presented as the median and interquartile range (IQR; Q1–Q3). Categorical variables were compared using Pearson’s chi-square test, the chi-square test with Yates’ continuity correction, or Fisher’s exact test, as appropriate. The strength of association was quantified by calculating odds ratios (ORs) with 95% confidence intervals (CIs).

Variables that demonstrated statistically significant differences between the restenosis and non-restenosis groups in the univariate analysis (*p* ≤ 0.05) were considered candidate predictors for multivariable analysis. A total of 23 variables met this criterion and were entered into the multivariable binary logistic regression model. Independent predictors of restenosis were identified using a forward stepwise likelihood ratio (Forward: LR) procedure. Variables were entered into the model at *p* < 0.05 and removed at *p* > 0.10 according to the default SPSS criteria. The final risk stratification model included only variables that remained independently associated with restenosis.

Model fit was assessed using the Nagelkerke R^2^ statistic. Model discrimination was evaluated using receiver operating characteristic (ROC) curve analysis by calculating the area under the ROC curve (AUC), sensitivity (Se), and specificity (Sp). Optimal cut-off values for continuous variables were determined using the Youden index.

To assess the robustness of the developed prediction model, internal validation was performed using bootstrap resampling with 1000 iterations. Model optimism was estimated by comparing the apparent and optimism-corrected model performance. Model calibration was evaluated using bootstrap-corrected calibration analysis, including the calibration slope, calibration intercept, maximum calibration error (Emax), and the Brier score. Calibration was further assessed using the Hosmer–Lemeshow goodness-of-fit test.

All statistical analyses were performed using StatTech version 4.9.5, IBM SPSS Statistics for Windows version 27.0 (IBM Corp., Armonk, NY, USA), and R version 4.5.x (R Foundation for Statistical Computing, Vienna, Austria) with the rms, Hmisc, and pROC packages.

## 3. Results

A total of 910 patients were included in the study, of whom 684 (75.2%) were male. The mean age was 62.9 ± 8.9 years (range, 32–88 years). The median follow-up time from the index PCI to follow-up coronary angiography was 14 months (IQR, 10–24 months). Participants were recruited from across Kazakhstan, with 41% residing in Astana and the Akmola region and 59% originating from other regions of the country. The proportion of ethnic Kazakhs to Caucasian individuals was approximately 3:1 (Table 1).

Among the studied patients, a history of MI increased the odds of coronary in-stent restenosis 2.2-fold (OR = 2.20; 95% CI: 1.65–2.80; *p* < 0.001). Type 2 diabetes mellitus was associated with a 2.6-fold higher risk of restenosis (OR = 2.60; 95% CI: 1.93–3.47; *p* < 0.001), while smoking increased the odds by 1.4 times (OR = 1.40; 95% CI: 1.01–1.89; *p* = 0.04).

Waist circumference was identified as a risk factor for cardiovascular disease. In the restenosis group, among 98 women, 93 (94.9%) had a waist circumference exceeding 88 cm, compared to 113 of 125 women (90.4%) in the non-restenosis group. Among 357 men in the restenosis group, 200 (56.0%) had a waist circumference exceeding 102 cm, whereas in the non-restenosis group, 174 of 330 men (52.7%) exceeded this threshold.

In the complete blood count analysis, statistically significant differences between the study groups were observed in white blood cell counts (WBC, *p* = 0.002), red blood cell distribution width-coefficient of variation (RDW-CV, *p* = 0.048), mean platelet volume (MPV, *p* < 0.001), platelet distribution width (PDW, *p* = 0.001), and absolute neutrophil count (ANC, *p* < 0.001), absolute monocyte count (AMC, *p* < 0.001) and absolute basophil count (ABC, *p* = 0.046). All of these parameters were significantly higher in the restenosis group.

Biochemical blood analysis revealed statistically significant differences in glucose (*p* < 0.001), triglycerides (*p* < 0.001), and HDL-C levels (*p* < 0.001). Compared with the non-restenosis group, patients with restenosis demonstrated higher glucose and triglyceride levels, along with lower HDL-C concentrations.

Furthermore, the calculated neutrophil-to-lymphocyte ratio, monocyte to high-density lipoprotein cholesterol ratio, atherogenic index, and triglyceride-glucose index were all significantly elevated in the restenosis group (*p* = 0.006; *p* < 0.001; *p* = 0.005; and *p* < 0.001, respectively).

No statistically significant differences were observed for other complete blood count or biochemical parameters between the groups (Table 2).

According to EchoCG data, LVEF was 52.7 [50.0; 56.2] in the restenosis group and 57.7 [54.7; 61.0] in the control group (*p* < 0.001).

Analysis of digital coronary angiography and intraoperative parameters demonstrated that multivessel CAD was more frequent in the restenosis group compared with the control group (83.1% vs. 67.3%, *p* < 0.001). A total of 1714 stents were implanted in 910 patients. The mean number of stents per patient was 2.3 in the case (restenosis) group and 1.5 in the control group (*p* < 0.001).

### Prediction of Coronary Artery Restenosis Risk in Patients with Coronary Artery Disease

Based on the literature review and the results of the univariate analysis, 23 variables (19 continuous and 4 dichotomous) were selected as candidate predictors and entered into the binary logistic regression analysis. To avoid multicollinearity, the variables with a high degree of mutual correlation (≥0.5) were not entered simultaneously into the regression model. The predictors were combined in different sequences across multiple modeling steps, and the final logistic regression equation was selected based on the highest overall predictive accuracy, which reached 74.4% for the study sample (Table 3).

The model’s goodness-of-fit, assessed using the Nagelkerke R^2^, was 0.405, indicating that approximately 40.5% of the variance in the outcome variable is explained by the logistic regression model.

The final logistic regression equation was as follows:

z = 0.829 X_1_ + 0.749 X_2_ + 0.487 X_3_ + 0.194 X_4_ − 0.918 X_5_ − 0.105 X_6_ − 0.577

where

X_1_—Type 2 diabetes mellitus (yes/no);

X_2_—Number of implanted coronary stents;

X_3_—MPV, fL;

X_4_—Absolute neutrophil count, 10^9^/L;

X_5_—HDL-C, mmol/L;

X_6_—LVEF, %;

−0.577—Constant.

The standard logistic regression equation used to calculate the model prediction was as follows:

$$P\;=\;\frac{1}{1+e^{-z}};$$

where P is the predicted probability of coronary in-stent restenosis, and z is the linear predictor calculated using the regression equation presented above.

The optimal probability threshold for classifying patients as having restenosis was 0.48, yielding an overall classification accuracy of 74.4%.

**Figure 1:**
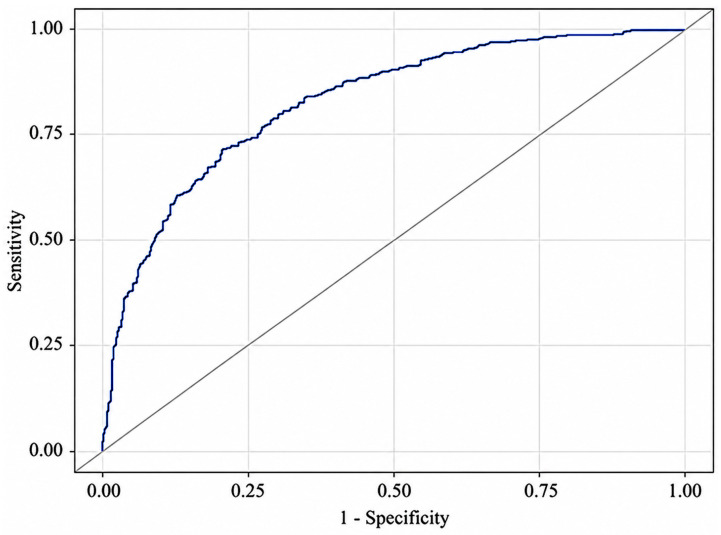
ROC curve of the coronary in-stent restenosis risk stratification model.

A ROC curve was constructed to illustrate the relationship between the true positive rate and the false positive rate for the binary classification results (Figure 1).

**Figure 2:**
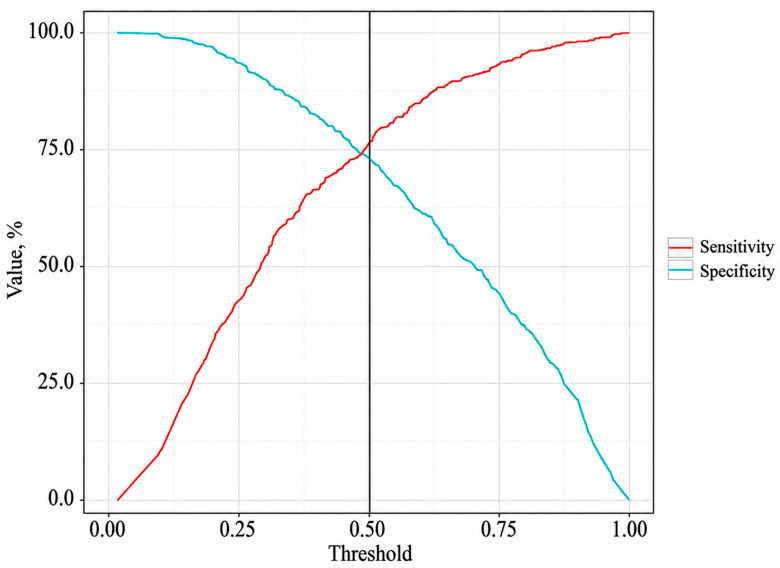
Sensitivity and specificity analysis of the model depending on the probability cut-off values for restenosis occurrence.

The area under the ROC curve was 0.828 ± 0.025 (95% CI: 0.802–0.855, *p* < 0.001). The model achieved an overall classification accuracy of 74.4%, with a sensitivity of 74.3% and a specificity of 74.5% (Figure 2).

Model calibration was first assessed using the Hosmer–Lemeshow goodness-of-fit test. The test indicated good agreement between predicted and observed outcomes (χ^2^ = 6.958, df = 8, *p* = 0.541), supporting adequate calibration of the prediction model.

The discriminative ability of the quantitative predictors included in the logistic regression model was further evaluated using ROC curve analysis. Optimal cut-off values were determined using the Youden index and were defined as the points providing the best balance between Se and Sp (Table 3).

Example of model application.

To illustrate the practical application of the proposed risk stratification model, the regression equation was applied to the following patient.

A 62-year-old man with type 2 diabetes mellitus underwent PCI with implantation of two coronary stents. His laboratory and echocardiographic parameters were as follows: MPV = 9.40 fL, absolute neutrophil count = 4.91 × 10^9^/L, HDL-C = 0.89 mmol/L, and LVEF = 40.71%.

The linear predictor was calculated as:

z = (0.829 × 1) + (0.749 × 2) + (0.487 × 9.40) + (0.194 × 4.91) − (0.918 × 0.89) − (0.105 × 40.71) − 0.577 = 2.189

Application of the regression equation to the patient’s data yielded the following value:

$$P\;=\;\frac{1}{1+e^{-2.189}}=0.899$$

As the calculated value exceeded the validated classification threshold of 0.48, this patient would be classified as belonging to the relatively higher-risk group for coronary in-stent restenosis according to the proposed risk stratification model.

Internal validation of the risk stratification model.

Internal validation was performed using bootstrap resampling with 1000 iterations to assess model stability and optimism. The apparent discrimination of the model (AUC = 0.828) remained essentially unchanged after optimism correction (optimism-corrected AUC = 0.825), indicating good internal validity and only limited optimism after internal bootstrap validation.

**Figure 3:**
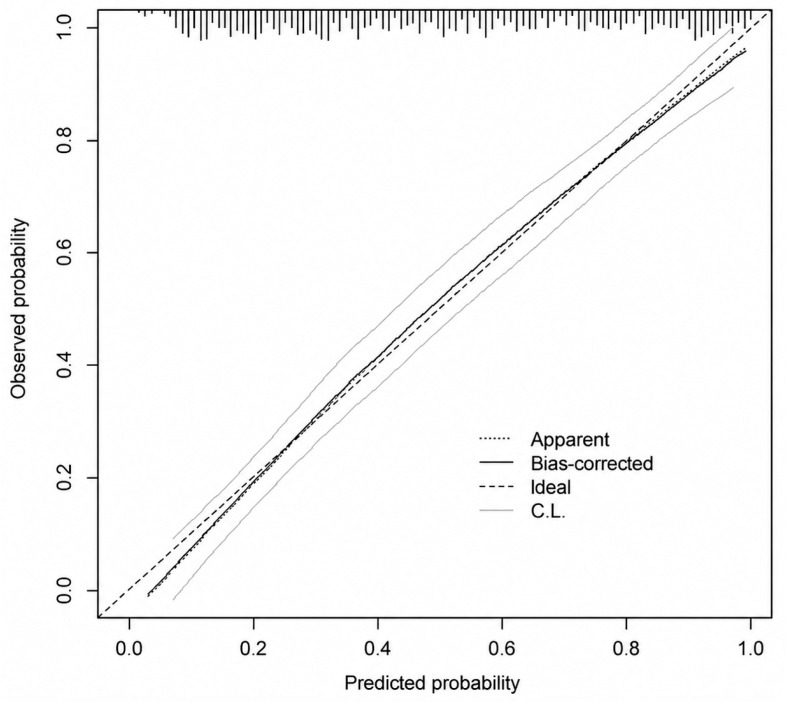
Bootstrap-corrected calibration plot of the prediction model for coronary in-stent restenosis.

Bootstrap-corrected calibration analysis demonstrated good agreement between predicted and observed probabilities. The optimism-corrected calibration slope was 0.980 (95% CI: 0.815–1.168), and the calibration intercept was 0.004 (95% CI: −0.191 to 0.211), indicating no evidence of systematic overestimation or underestimation of risk. The maximum calibration error (Emax) was 0.026, and the optimism-corrected Brier score was 0.172. The bootstrap-corrected calibration plot further confirmed good agreement between predicted and observed risks across the full range of predicted probabilities (Figure 3).

## 4. Discussion

In the present study, independent predictors of coronary in-stent restenosis were identified based on the analysis of routinely available clinical and laboratory parameters included in national diagnostic protocols for patients with CAD in the Republic of Kazakhstan. The identified predictors reflect complementary aspects of restenosis pathophysiology, including patient-related clinical characteristics, inflammatory and metabolic laboratory markers, and procedural factors associated with PCI. Based on these findings, a clinically applicable risk stratification model incorporating six independent predictors was developed and internally validated, demonstrating good discrimination, satisfactory calibration, and limited optimism after internal bootstrap validation.

A longer history of coronary artery disease may represent an important risk factor for the development of coronary in-stent restenosis following PCI. Although no studies have directly evaluated the duration of CAD as an independent predictor, accumulating evidence suggests that markers of a chronic disease course are associated with an increased risk of ISR. A prolonged history of coronary artery disease likely reflects a greater and more diffuse atherosclerotic burden, accompanied by persistent low-grade inflammation and structural alterations of the vascular wall, including progressive calcification, fibrosis and heterogeneous vascular remodeling. Collectively, these factors may increase the likelihood of both mechanical suboptimal stent deployment (e.g., underexpansion or incomplete apposition) and the activation of maladaptive reparative processes, ultimately leading to neointimal hyperplasia and restenosis.

A history of MI represents a significant risk factor for the development of restenosis. According to a recent systematic review and meta-analysis, prior myocardial infarction is identified as a well-established independent predictor of in-stent restenosis, with an odds ratio of 1.79 (95% CI: 1.12–2.86) [10].

Smoking was associated with a 1.4-fold increase in the risk of coronary in-stent restenosis, which is consistent with findings from previous studies (RR = 1.63; 95% CI: 1.25–2.13). This observation further supports the role of smoking as a significant risk factor and is in line with its established contribution to enhanced oxidative stress, pro-inflammatory activity and accelerated neointimal hyperplasia [11,12].

In our cohort, no significant differences were observed between patients with and without restenosis in terms of body mass index (BMI) or the presence of general obesity, which is consistent with findings from large-scale clinical studies. Specifically, the TAXUS-IV trial demonstrated that obesity was associated with a higher risk of restenosis and major adverse cardiovascular events in patients treated with BMS, whereas this excess risk was markedly attenuated with the use of paclitaxel-eluting stents [13,14]. Consequently, mid-term outcomes after PCI with drug-eluting stents appear to be largely independent of overall body mass. Importantly, our results extend these observations by demonstrating a significant association between waist circumference and restenosis, supporting the concept that abdominal adiposity, rather than generalized obesity, represents a more relevant determinant of adverse vascular remodeling after stent implantation. This finding supports the importance of incorporating measures of visceral fat distribution into post-PCI risk stratification, beyond conventional BMI-based assessment [15].

Recent meta-analyses consistently demonstrate that diabetes mellitus is associated with a 46–59% increase in the risk of restenosis. This effect has been attributed to hyperglycemia-driven mechanisms, including enhanced neoangiogenesis and chronic vascular inflammation, which promote accelerated neointimal hyperplasia [10,16]. The findings of the present study are in line with these observations, further supporting the role of diabetes as a key determinant of restenosis after coronary stent implantation.

Laboratory analyses revealed that patients with restenosis exhibited higher levels of total WBC, neutrophils, monocytes, and basophils, reflecting an enhanced systemic inflammatory response. Elevated absolute neutrophil counts and increased NLR have been associated with a higher risk of in-stent restenosis, as neutrophils play a key role in the early phases of vascular wall inflammation following stent implantation and in neointimal remodeling. These observations are supported by multiple studies identifying NLR as a prognostic marker of restenosis [17,18,19].

Elevated RDW-CV was recognized as an independent predictor of in-stent restenosis even during the era of BMS. RDW-CV reflects the variability in erythrocyte size and is sensitive to inflammatory and metabolic disturbances that promote neointimal hyperplasia. Although the advent of DES was expected to reduce the predictive value of this marker, recent studies indicate that RDW-CV retains its prognostic significance with both first- and second-generation DES [20,21].

Platelet indices, including MPV and PDW, have been investigated as predictors of restenosis. Elevated MPV reflects the presence of larger, more reactive platelets with pronounced prothrombotic and pro-inflammatory potential, while increased PDW indicates platelet heterogeneity and activation. Several studies have demonstrated that both indices are associated with a higher risk of in-stent restenosis, particularly among patients with coronary artery disease and diabetes mellitus [22,23,24].

Biochemical analysis of lipid metabolism markers in patients with restenosis revealed lower levels of HDL-C and elevated triglyceride levels, reflecting an adverse lipid profile associated with enhanced inflammation and endothelial dysfunction. HDL-C possesses well-established anti-inflammatory and anti-atherogenic properties, and its reduction may facilitate activation of vascular wall inflammatory responses. Conversely, elevated triglycerides are linked to metabolic stress and accelerated atherogenesis. A significant increase in the MHR, which combines markers of inflammation and dyslipidemia, further indicates a heightened inflammatory state in patients with restenosis, suggesting that MHR may serve as a potential indicator of recurrent stent-related events [25,26].

Additional metabolic markers reflecting the risk of vascular complications include the atherogenic index and the TyG index. The atherogenic index represents the ratio of atherogenic to anti-atherogenic lipoproteins and serves as an integrated measure of dyslipidemia severity; elevated values are associated with accelerated atherogenesis and endothelial dysfunction. The TyG index, calculated from fasting triglyceride and glucose levels, is considered a reliable surrogate marker of insulin resistance and metabolic stress. Higher TyG values are linked to enhanced inflammatory responses, progression of atherosclerosis, and increased risk of adverse cardiovascular outcomes, supporting the role of metabolic dysregulation in the development of restenosis [27]. Recent evidence suggests that the prognostic significance of glucometabolic dysregulation extends beyond diabetes mellitus and the TyG index. In particular, the stress hyperglycemia ratio (SHR), reflecting acute glycemic stress relative to chronic glycemic status, has been associated with PCI-related myocardial complications and adverse cardiovascular outcomes in patients with acute myocardial infarction. These findings further highlight the important role of metabolic disturbances in post-PCI risk assessment [28].

Left ventricular ejection fraction is a well-established prognostic marker in patients with cardiovascular diseases. Reduced LVEF is associated with worse clinical outcomes, including progression of heart failure and increased cardiovascular mortality. Conversely, higher LVEF reflects a more favorable cardiovascular profile and may indirectly indicate a lower likelihood of adverse events following PCI [10,29].

In addition to patient-related factors, previous studies have shown that anatomical lesion characteristics and procedural complexity are associated with the risk of restenosis. Diffuse coronary artery disease and a greater number of implanted stents have been associated with an increased likelihood of recurrent stenosis. While diffuse coronary artery disease reflects a greater atherosclerotic burden, a higher number of implanted stents may increase endothelial injury and local inflammatory responses following PCI [10,16].

Unlike several previously published ISR prediction models that incorporate detailed angiographic characteristics such as lesion length, vessel diameter, and stent dimensions, the present model was intentionally developed using routinely available clinical, laboratory, and procedural factors. Although this approach limits direct comparison with models based on detailed quantitative coronary angiography, it enhances the applicability of the proposed model in routine clinical practice, where such information may not be readily available during patient risk assessment.

One important consideration is that the prediction model was developed using a balanced retrospective case–control design. Although the model demonstrated good discrimination and calibration within the study population, the predicted probabilities should be interpreted primarily for relative risk stratification rather than as estimates of absolute risk in the general PCI population. Accordingly, the model should be regarded as a tool for identifying patients at relatively higher likelihood of restenosis who may benefit from closer clinical surveillance and further diagnostic evaluation. Given that only internal validation was performed, external validation and, if necessary, recalibration in independent cohorts reflecting the natural prevalence of restenosis are required before widespread clinical implementation.

### Limitations

The present study has several limitations. First, it used a retrospective design. The duration of coronary disease was estimated based on medical records; however, the exact onset of the disease could not be reliably established. Asymptomatic patients who did not undergo subsequent coronary angiography were not captured, which may have resulted in under-ascertainment of clinically silent restenosis cases.

Second, the study did not account for the types and classifications of implanted drug-eluting stents, limiting our ability to evaluate the impact of different stent models on the development of in-stent restenosis.

Third, the model was developed using a balanced retrospective case–control design with equal numbers of patients with and without restenosis. Therefore, the predicted probabilities should be interpreted primarily as a tool for patient risk stratification rather than as estimates of the absolute risk of restenosis in the general PCI population. External validation and, if necessary, recalibration in cohorts reflecting the natural prevalence of restenosis are required before broad clinical implementation.

Fourth, several well-established angiographic and procedure-related predictors of in-stent restenosis, including reference vessel diameter, lesion length, total stent length, and stent diameter, were not systematically available for the entire cohort. These variables are recognized predictors of restenosis and have been incorporated into several previously published prediction models. Their absence limits direct comparison between the present model and models incorporating detailed angiographic characteristics. Nevertheless, the proposed model was intentionally developed using routinely available clinical, laboratory, and procedural factors to maximize its applicability in routine clinical practice.

Fifth, although information on prescribed pharmacological therapy was available from the medical records, complete longitudinal information regarding medication adherence, treatment modifications during follow-up, and duration of therapy was not consistently available for the entire cohort because of the retrospective study design and variable intervals between the index PCI and follow-up coronary angiography. Consequently, medication-related variables were not included in the multivariable model and may have contributed to residual confounding. Future prospective studies with comprehensive longitudinal medication data are warranted to further validate and refine the proposed risk stratification model.

Sixth, repeat coronary angiography was clinically driven or prompted by complex index procedures rather than performed as routine surveillance. This approach may have introduced selection bias, since patients with silent restenosis or those at lower risk were less likely to undergo follow-up imaging. Therefore, the study findings primarily reflect patients with clinical indications for repeat angiography.

## 5. Conclusions

Coronary in-stent restenosis remains an important challenge in contemporary interventional cardiology, highlighting the need for reliable tools to identify patients at increased risk following PCI. In the present study, we developed and internally validated a risk stratification model based on routinely available clinical, laboratory, and procedural factors, including type 2 diabetes mellitus, the number of implanted coronary stents, mean platelet volume, absolute neutrophil count, HDL-C, and left ventricular ejection fraction.

The proposed model demonstrated good discriminative performance (AUC = 0.828) and satisfactory internal validity with limited optimism after internal bootstrap validation. It may serve as a useful tool for patient risk stratification after PCI.

Because the model was developed using a balanced retrospective case–control design and underwent internal validation only, its predictions should be interpreted primarily for relative risk stratification rather than as estimates of absolute risk in the general PCI population. External validation and, if necessary, recalibration in independent cohorts reflecting the natural prevalence of restenosis are required before widespread clinical implementation.

## Figures and Tables

**Table 1:** Baseline patient characteristics.

Characteristic/Parameter	All Patients *n* = 910	Restenosis Group *n* = 455	Non-Restenosis Group *n* = 455	*p*
Age, years	62.89 ± 8.853	62.71 ± 8.460	63.07 ± 9.236	0.537
Gender male/ female, *n* (%)	684/226 (75.2/24.8)	354/101 (77.8/22.2)	330/125 (72.5/27.5)	0.066
Ethnic Kazakhs/Caucasian, *n* (%)	672/238 (73.8/26.2)	334/121 (73.4/26.6)	338/117 (74.3/25.7)	0.763
Duration of coronary disease, months	60.0 (24.0–108.0)	74.0 (36.0–120.5)	36.0 (18.0–72.0)	<0.001
History of MI, *n* (%)	462 (50.8)	274 (60.2)	188 (41.3)	<0.001
Smoking, *n* (%)	208 (22.9)	117 (25.7)	91 (20.0)	0.040
Family history of cardiovascular disease, *n* (%)	229 (25.2)	120 (26.4)	109 (24.0)	0.401
Obesity, *n* (%)	559 (61.4)	285 (62.6)	274 (60.2)	0.454
Waist circumference, cm	103.0 (97.0–109.0)	104.0 (98.0–110.0)	102.0 (96.0–107.6)	0.002
Type 2 diabetes mellitus, *n* (%)	277 (30.4)	183 (40.2)	94 (20.7)	<0.001

**Table 2:** Summary of Complete Blood Count and Biochemical Parameters.

Parameter	All Patients *n* = 910	Restenosis Group *n* = 455	Non-Restenosis Group *n* = 455	*p*
WBC, 10^9^/L	6.56 [5.54; 7.70]	6.73 [5.63; 7.97]	6.40 [5.47; 7.48]	0.002
RDW-CV, %	13.3 [12.9; 14.0]	13.4 [12.9; 14.2]	13.3 [12.8; 13.9]	0.048
MPV, fL	10.0 [9.4; 10.8]	10.3 [9.6; 11.3]	9.9 [9.2; 10.5]	<0.001
PDW, fL	11.4 [10.3; 12.8]	11.7 [10.4; 13.1]	11.2 [10.1; 12.5]	0.001
ANC, 10^9^/L	3.91 [3.14; 4.80]	4.07 [3.23; 5.06]	3.79 [3.06; 4.61]	<0.001
AMC, 10^9^/L	0.52 [0.42; 0.62]	0.55 [0.44; 0.63]	0.50 [0.40; 0.61]	<0.001
ABC, 10^9^/L	0.03 [0.02; 0.05]	0.03 [0.02; 0.05]	0.03 [0.02; 0.04]	0.046
Glucose, mg/dL	102.29 [93.52; 120.78]	105.80 [95.45; 129.70]	99.67 [91.95; 113.05]	<0.001
Triglycerides, mmol/L	1.45 [1.02; 2.08]	1.56 [1.07; 2.30]	1.37 [1.01; 1.87]	<0.001
HDL-C, mmol/L	1.11 [0.96; 1.30]	1.08 [0.94; 1.26]	1.16 [0.98; 1.33]	<0.001
NLR	2.09 [1.65; 2.74]	2.18 [1.71; 2.86]	2.01 [1.62; 2.67]	0.006
MHR	0.46 [0.35; 0.61]	0.49 [0.38; 0.64]	0.43 [0.33; 0.58]	<0.001
Atherogenic index	3.07 [2.23; 4.11]	3.21 [2.33; 4.17]	2.97 [2.14; 3.98]	0.005
TyG index	8.83 [8.48; 9.25]	8.94 [8.53; 9.43]	8.74 [8.41; 9.09]	<0.001

**Table 3:** Results of stepwise logistic regression analysis.

Step	Variable	Wald χ^2^	Regression Coefficient (β)	OR	95% CI	*p*	Cut-Off
1	Type 2 diabetes mellitus	22.233	0.829	2.290	1.623; 3.231	<0.001	
2	Number of implanted coronary stents	69.178	0.749	2.115	1.773; 2.524	<0.001	2
3	MPV	42.484	0.487	1.627	1.406; 1.884	<0.001	10.0
4	Absolute neutrophil count	12.617	0.194	1.214	1.091; 1.352	<0.001	3.9
5	HDL-C	10.878	−0.918	0.399	0.231; 0.689	0.001	1.1
6	LVEF	63.985	−0.105	0.900	0.877; 0.924	<0.001	55.0
7	Constant		−0.577			0.616	

## Data Availability

The data presented in this study are available on reasonable request from the corresponding author. The data are not publicly available due to privacy and ethical restrictions, as they contain de-identified patient-level clinical data and are subject to institutional approval.

## Abbreviations

The following abbreviations are used in this manuscript:

ABC	Absolute basophil count
AMC	Absolute monocyte count
ANC	Absolute neutrophil count
AUC	Area under the curve
BMI	Body mass index
BMS	Bare-metal stents
CAD	Coronary artery disease
CI	Confidence Interval
DES	Drug-eluting stents
EchoCG	Echocardiography
HDL-C	High-density lipoprotein cholesterol
ISR	In-stent restenosis
LVEF	Left ventricular ejection fraction
MHR	Monocyte to high-density lipoprotein cholesterol ratio
MI	Myocardial infarction
MPV	Mean platelet volume
NLR	Neutrophil-to-lymphocyte ratio
OR	Odds ratio
PCI	Percutaneous coronary intervention
PDW	Platelet distribution width
RDW-CV	Red blood cell distribution width-coefficient of variation
ROC	Receiver operating characteristic
Se	Sensitivity
SHR	Stress hyperglycemia ratio
Sp	Specificity
T2DM	Type 2 diabetes mellitus
TyG index	Triglyceride-glucose index
WBC	White blood cell
